# Non-vitamin K antagonist oral anticoagulants in adults with a Fontan circulation: are they safe

**DOI:** 10.1136/openhrt-2018-000985

**Published:** 2019-06-03

**Authors:** Hayang Yang, Gruschen R Veldtman, Berto J Bouma, Werner Budts, Koichiro Niwa, Folkert Meijboom, Giancarlo Scognamiglio, Alexander Chima Egbe, Markus Schwerzmann, Craig Broberg, Marielle Morissens, Jonathan Buber, Shane Tsai, Ioannis Polyzois, Martijn C Post, Matthias Greutmann, Arie Van Dijk, Barbara JM Mulder, Jamil Aboulhosn

**Affiliations:** 1 Deparment of Cardiology, AMC, Amsterdam, The Netherlands; 2 Adolescent and Adult Congenital Heart Disease Program, Cincinnati Children's Hospital Medical Centre, Cincinnati, Ohio, USA; 3 Cardiology, Academical Medical Center-University of Amsterdam, Amsterdam, The Netherlands; 4 Department of Cardiology, University Hospital Gasthuisberg, Leuven, Belgium; 5 Deparment of Cardiology, St Luke's International Hospital, Tokyo, Japan; 6 Cardiology, University Medical Centre Utrecht, Utrecht, Netherlands; 7 Cardiology, Monaldi Hospital-Second University of Naples, Naples, Italy; 8 Deparment of Medicine, Division of Cardiovascular Diseases, Mayo Clinic, Rochester, New York, USA; 9 Adult Congenital Heart Diseae Program, University Hospital Inselspital, Bern, Switzerland; 10 Knight Cardiovascular Institute, Oregon Health & Sciences University, Portland, Oregon, USA; 11 Department of Cardiology, CHU Brugmann, Brussels, Belgium; 12 Heart Center, Sheba Medical Center, Ramat Gan, Israel; 13 Department of Cardiology, University of Nebraska Medical Centre, Omaha, Nebraska, USA; 14 Adult Congenital Heart Centre and Centre for Pulmonary Hypertension, Royal Brompton Hospital, London, UK; 15 Department of Cardiology, St Antonius Hospital, Nieuwegein, The Netherlands; 16 Cardiology, University Hospital Zurich, Zurich, Switzerland; 17 Cardiology, Nijmegen University Medical Center, Nijmegen, The Netherlands; 18 Cardiology, Academic Medical Center, Amsterdam, The Netherlands; 19 Cardiology, UCLA, Los Angeles, California, USA

**Keywords:** fontan circulation, adult congenital heart disease, anticoagulation, thromboembolism, bleeding

## Abstract

**Background:**

In Fontan patients with atrial arrhythmias (AA), non-vitamin K antagonist oral anticoagulants(NOACs) have a class III recommendation according to the Pediatric & Congenital Electrophysiology Society (PACES)/Heart Rhythm Society (HRS) guideline in 2014, due to lack of data on outcomes as opposed to evidence of harm. To address this gap in data, we investigated the safety and efficacy of NOACs in adults with a Fontan circulation in a worldwide study.

**Methods:**

This is an international multicentre prospective cohort study, using data from the NOTE (**n**on-vitamin K antagonist **o**ral anticoagulants for **t**hrombo**e**mbolic prevention in patients with congenital heart disease) registry. The study population comprised consecutive adults with a Fontan circulation using NOACs. Follow-up took place at 6 months and yearly thereafter. The primary endpoints were thromboembolism and major bleeding. Secondary endpoint was minor bleeding.

**Results:**

From April 2014 onward, 74 patients (mean age 32±10 years (range 18–68), 54% male) with a Fontan circulation using NOACs were included. During a median follow-up of 1.2 (IQR 0.8–2.0) years, three thromboembolic events (2.9 per 100 patient-years (95% CI 0.7 to 7.6)) and three major bleedings (2.9 per 100 patient-years (95% CI 0.7 to 7.6)) occurred in five atriopulmonary Fontan and one total cavopulmonary connection Fontan patients with AA. Fifteen patients experienced minor bleeding episodes (15.8 per 100 patient-years (95% CI 9.1 to 25.2)). In patients (n=37) using vitamin K antagonists (VKAs) prior to the initiation of NOAC, annual incidence of historical thromboembolic events and major bleeding were 2.4% (95% CI 0.4% to 7.4%) (n = 2) and 1.2% (95% CI 0.7% to 5.1%) (n = 1), respectively.

**Conclusions:**

In this review of the largest Fontan cohort using NOACs with prospective follow-up, NOACs appear to be well tolerated and their efficacy and safety during short-term follow-up seem comparable to VKAs. Longer term data are required to confirm these promising short-term results.

**Key questions**

**What is already known about this subject?**

- In Fontan patients with atrial arrhythmias, non-vitamin K antagonist oral anticoagulants (NOACs) have a class III recommendation according to the PACES/HRS guideline in 2014, despite lack of data on outcomes as opposed to evidence of harm.

**What does this study add?**

- This international multicentre prospective cohort study shows that NOACs seem to be well tolerated and have comparable efficacy and safety as vitamin K antagonists during short-term follow-up in adults with a Fontan circulation.

**How might this impact on clinical practice?**

- With growing evidence of efficacy and safety results with NOACs, future guidelines should reassess NOACs as a treatment option for prevention of thromboembolism in Fontan patients.

## Introduction

Patients with a Fontan circulation have a paradoxical coexistence of an increased risk of thromboembolism as well as bleeding.^1–3^ However, optimal thromboprophylaxis is unclear in this patient group due to limited evidence for efficacy and safety of anticoagulation or antiplatelet treatment, mostly consisting of retrospective studies performed in children or mixed cohorts with adult patients.^4–7^ Furthermore, conventional thromboprophylaxis such as vitamin K antagonists (VKAs) and aspirin have important limitations since they are suboptimal with considerable residual thromboembolic risk of 9.8%–13%,^3^ while there are difficulties achieving consistent international normalised ratio (INR) range with VKAs,^4^ and 52% of aspirin-treated Fontan adults having aspirin resistance.^5^ Also, pursuit of optimal thromboprophylaxis continues to be a growing concern as the numbers of adults with Fontan circulation are growing with future prospect of more than 50% of these patients acquiring atrial arrhythmias (AA) at 20 years after a Fontan procedure.^6^

Non-vitamin K antagonist oral anticoagulants (NOACs) are an attractive alternative to VKAs with the potential for reduced intracerebral haemorrhage, relatively few dietary or drug interactions and no need for ongoing INR monitoring as a result of their predictable pharmacokinetics. However, in Fontan patients with AA, NOACs have a class III recommendation according to the recent PACES/HRS guideline in 2014,^7^ due to lack of data on outcomes as opposed to evidence of harm. Hence, providers were initially wary of prescribing NOACs in this population due to concerns over lack of data to support efficacy and safety. However, in the course of time, despite the class III recommendation, NOACs are increasingly being used in clinical practice. Therefore, there is growing need to obtain safety and efficacy data of NOACs in Fontan patients.

To address this gap in data and to monitor the application of NOACs in daily clinical practice, we investigated the safety and efficacy of NOACs in adults with a Fontan circulation in a worldwide prospective observational study, by means of the NOTE registry: NOACs for thromboembolic prevention in patients with congenital heart disease.^8^

## Materials and methods

### Study design and participants

NOTE is an ongoing international and multicentre prospective registry of adults with congenital heart disease (ACHD) on NOACs for the prevention of thromboembolism. All individual participants gave informed consent in accordance with national and local regulations. Through collaboration with the International Society for Adult Congenital Heart Disease, patients were recruited from April 2014 in 35 centres from 10 countries distributed over various continents including Europe, North America, Middle East and East Asia. The patients were included as they presented at the participating institutions or were identified by using a national registry. From the NOTE registry, all consecutive Fontan patients were identified and included. At baseline and follow-up, anatomic and clinical data including the details on anticoagulation use were collected. VKA-experienced group and aspirin-experienced group consisted of the patients who used VKA or aspirin at the time of transitioning to NOAC, respectively. Prospective follow-up took place at 6 months or/and 1 year, and 2 years, coinciding with their routine outpatient clinical visit or telephone contact. At such follow-up point, evaluation for predefined end points was conducted by an investigator or a site coordinator, who was trained on the protocol of the NOTE registry.

### Data management

We collected patient data in an electronic case report form (eCRF) and submitted on a secure website, which is certified for Good Clinical Practice. We assigned all patients with a unique study identifier so that personal identifiable data could be removed at the hospital source, ensuring anonymity and protecting confidentiality. The principle registry coordinator and each site coordinator examined the completeness and accuracy of the eCRFs. The principle registry coordinator as an author had full access to all the data in the study and takes responsibility for its integrity and the data analysis.

### Study outcomes

The primary endpoints were thromboembolism (ischaemic cerebrovascular accident, transient ischaemic attack, systemic or pulmonary embolism or intracardiac thrombosis) and major bleeding (significant bleeding necessitating hospitalisation/interventions/≥2 units of packed cells and/or with a haemoglobin drop >1.24 mmol/L (20g/L) and/or bleeding that was fatal or occurred in the following critical sites: intracranial, intraspinal, intraocular, pericardial, intra-articular, intramuscular with compartment syndrome) according to the International Society on Thrombosis and Haemostasis criteria.^9^ Secondary endpoint was minor bleeding, defined as any bleeding that could not be classified as major bleeding.

### Statistical analysis

Descriptive statistics were used to delineate baseline characteristics. Incidence rates of the outcomes were calculated using Poisson regression. Kaplan-Meier survival curves were created to determine event-free survival. Similarly, incidence rates and event-free survival under VKA or aspirin treatment were determined using the historical data of VKA-experienced and aspirin-experienced patients for reference. Results are presented as mean with SD (±), median with IQR and incidence rates with 95% CIs where appropriate. Analyses were performed using R V.3.2.4 (R core team) and SPSS V.23 (IBM).

## Results

From April 2014 onward, 74 patients (mean age 32±10 years (range 18–68), 54% male) with a Fontan circulation were identified from 513 ACHD on NOACs (anabaptist, n=27; dabigatran, n=7; edoxaban, n=4; rivaroxaban, n=36) in the NOTE registry (table 1). The indication for NOACs was AA in 52 patients, primary prevention of thromboembolism in 12 patients and secondary prevention of thromboembolism in 10 patients. CHA2DS2-VASc score was ≥1 in 49 patients (66%) and only 2 patients had a high HAS-BLED (bleeding risk factor scoring system in which 1 point is given for uncontrolled hypertension, abnormal renal or liver function, history of stroke or bleeding, labile international normalized ratio, age>65 years, use of nonsteroidal anti-inflammatory drug or antiplatelet agents or alcohol) score of t3.

**Table 1:** Baseline characteristics of adults with Fontan circulation using non-vitamin K antagonist oral anticoagulants (NOACs)

	All (n=74)
Age at inclusion, year	32±10
Male, n (%)	40 (54)
Congenital heart defect, n (%)	
Tricuspid atresia	27 (36)
Pulmonary atresia	10 (14)
Double outlet right ventricle	11 (15)
Double inlet left ventricle	14 (19)
Other anomalies	12 (16)
Type of Fontan, n (%)	
Atriopulmonary	26 (35)
Total cavopulmonary connexion	48 (65)
Previous antithrombotic medication, n (%)	
None	18 (24)
Vitamin K antagonist	37 (50)
Aspirin	19 (26)
Indication for NOAC, n (%)	
Atrial arrhythmias	52 (70)
Primary thrombotic prophylaxis	12 (16)
Secondary thrombotic prophylaxis	10 (14)
Median CHA_2_DS_2_-VASc	1 (0–2)
Median HASBLED	0 (0–1)
Cardiovascular history, n (%)	
Stroke or transient ischaemic attack (TIA)	8 (11)
Pulmonary embolism	4 (5)
Deep venous thrombosis	1 (1)
Intracardiac thrombosis	7 (9)
Inferior vena cava thrombosis	4 (5)
Superior vena cava thrombosis	1 (1)

Values are presented as mean (±), median (IQR) or counts (%).

CHA2DS2-VASc, stroke risk factor scoring system in which 1 point is given for heart failure, hypertension, age 64–74 years, diabetes mellitus, history of vascular disease, female sex and 2 points are given for age ≥75 years, history of stroke/TIA/thromboembolism; HASBLED, bleeding risk factor scoring system in which 1 point is given for uncontrolled hypertension, abnormal renal or liver function, history of stroke or bleeding, labile international normalised ratio, age >65 years, use of nonsteroidal anti-inflammatory drug or antiplatelet agents or alcohol.

**Figure 1:**
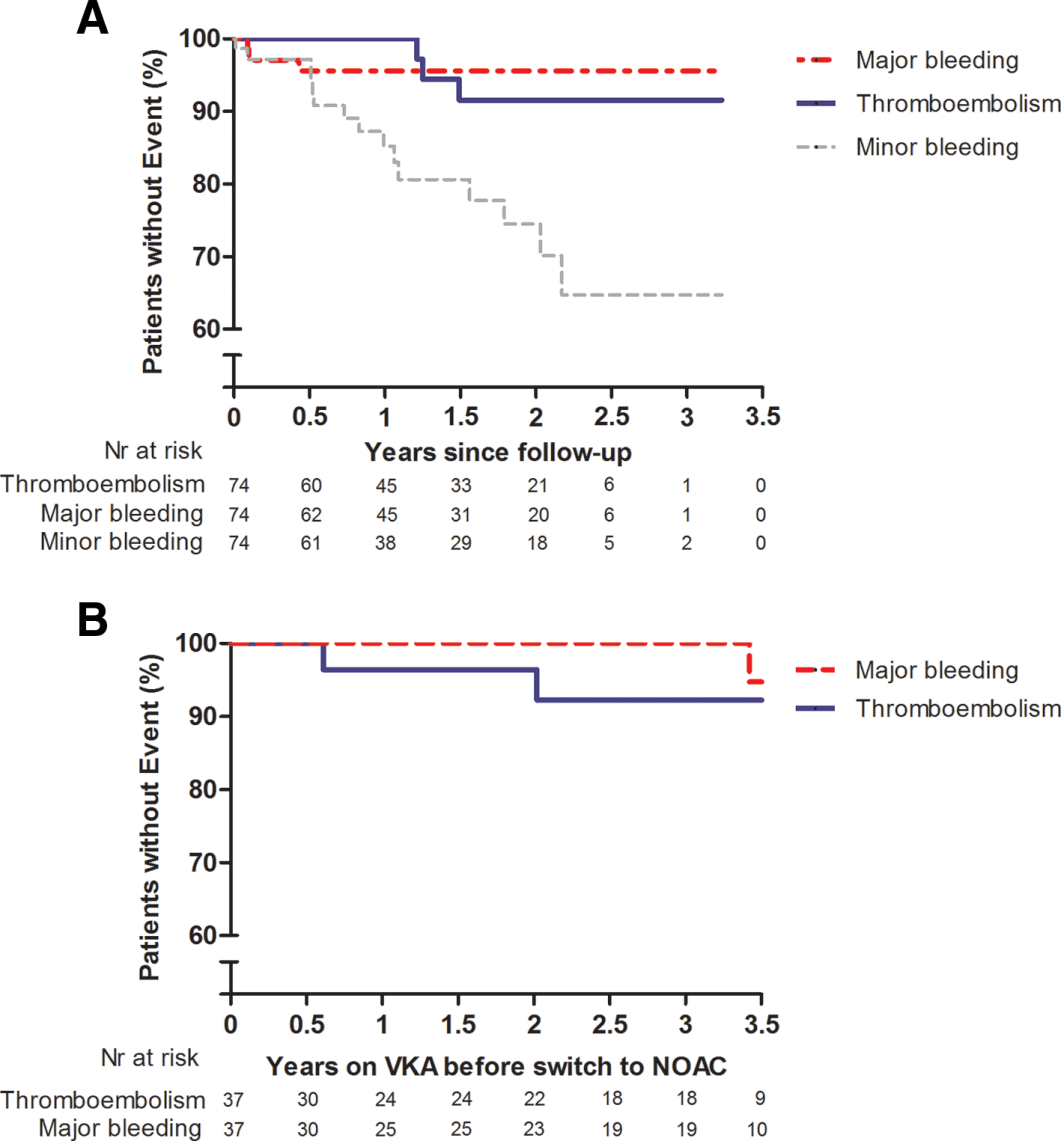
(A) Kaplan-Meier curves for survival free from thromboembolic event, major bleeding and minor bleeding in adult Fontan patients using NOACs. Nr, number. (B). Kaplan-Meier estimates of thromboembolic event and major bleeding in adult Fontan patients using VKA during 3.5 years, previous to NOACs use. NOAC, non-vitamin K antagonist oral anticoagulant; VKA, vitamin K antagonist.

During a mean follow-up of 1.4±0.9 years (total follow-up period 102.4 patient-years), in total, three thromboembolic events (two pulmonary embolisms, one ischaemic stroke; annual incidence 2.9% (95% CI 0.7% to 7.6%)), and three major bleedings (two menorrhagia, one major gastrointestinal bleeding; annual incidence 2.9% (95% CI 0.7% to 7.6%)) occurred in five atriopulmonary Fontan patients and one total cavopulmonary connection Fontan patient, all with AA (figure 1A, table 2). Fifteen patients experienced a minor bleeding (annual incidence 15.8% (95% CI 9.1% to 25.2%)) (figure 1A, table 2). During follow-up, two patients died, one due to heart failure and one due to cancer. In total, 14 patients ceased NOACs and resumed on VKAs due to thromboembolic event (n=3), bleeding (n=4), side effects (vasculitis, headache, n=2), pregnancy (n=2), patient refusal (n=2) and 1 following Fontan conversion. A subgroup of patients, who had no antithrombotic medication prior to starting NOACs and used NOACs for primary prevention of thromboembolism (n=4), did not experience any thromboembolic event or major bleeding during follow-up. From this subgroup, one patient experienced minor bleeding (menorrhagia).

**Table 2:** All bleeding events under non-vitamin K antagonist oral anticoagulants

Type of bleeding, n (%)	All patients (n=74)
Major bleeding	
Total	3
Menorrhagia	2
Gastrointestinal bleeding	1
Minor bleeding	
Total	15
Menorrhagia	6
Skin haematoma	3
Epistaxis	2
Bleeding leading to change in antithrombotic therapy	2
Prolonged bleeding after minor cut	1
Gingival bleeding	1

Prior to the initiation of NOAC, 50% (n=37) of the patients used VKAs, 26% (n=19) aspirin and 24% (n=18) no antithrombotic medication. During a period of 3.5 years prior to NOAC initiation, annual incidence of historical thromboembolic events and major bleeding were 2.4% (95% CI 0.4% to 7.4%) (n=2) and 1.2% (95% CI 0.7% to 5.1%) (n=1), respectively, in patients previously using VKAs (n=37) (figure 1B). In patients previously using aspirin (n=19), annual incidence of historical thromboembolic events and major bleeding were 6.2% (95% CI 1.9% to 14.4%) (n=4) and 0% (95% CI 0% to 0%) (n=0), respectively. The incidence of minor bleeding under the historical use of VKAs and aspirin could not reliably be retrieved from the medical files retrospectively.

## Discussion

This is the first study to prospectively evaluate data on safety of NOACs in 70 adults with a Fontan circulation included in a worldwide setting. We demonstrate an annual incidence of 2.9% thromboembolism, 2.9% major bleeding and 15.8% minor bleeding in Fontan patients actively using NOACs.

Our results offer important insight to NOACs’ potential in thromboembolic prevention in Fontan patients. Although limited by study design and smaller number of participants, a retrospective study in 21 adult Fontan patients (12 with AA) showed few adverse events (1 thromboembolic event, no major bleeding, median follow-up 13 months) and therefore also did not raise safety concerns in using NOACs in Fontan adults in short term.^10^ In contrast with NOACs, many retrospective studies have investigated thromboembolic event and to a much smaller extent major bleeding in Fontan patients using VKAs or antiplatelet therapy.^11^ However, most of these studies were done in mixed cohort of paediatric and adult patients and did not clearly present the annual rates of thromboembolic and major bleeding events for comparison. One recently published retrospective study by Faircloth *et al* showed annual incidence of 1.9% thromboembolism, 3.8% major bleeding and 30.2% minor bleeding in 45 Fontan patients (median age 19 years, 29% AA) using VKAs for 19 months.^4^ Only two recently published retrospective studies by Egbe *et al* determined thromboembolic and major bleeding events specifically in adult Fontan population using antithrombotic therapy.^3 4^ One of his studies showed an annual incidence of 5.2% thromboembolic event in 387 adult Fontan cohort (mean age 28 years, 72% AA) largely using aspirin (85%) or warfarin (14%) with mean follow-up of 8 years.^12^ Unfortunately, major bleeding (8 events including 2 intracranial haemorrhage) and minor bleeding events (21 events) were not reported as incidence rates to be compared with our cohort using NOACs. Egbe *et al*’s other study showed annual incidence of 6.5% thromboembolism and 0.4% major bleeding in 278 adult Fontan cohort with AA (mean age 31 years) largely using antiplatelet therapy (65%) or anticoagulation therapy (33%, of which 97% warfarin, 3% rivaroxaban), of which 64% used additional antiplatelet therapy.^2^ As illustrated, it remains challenging to compare our results to previous studies in adult Fontan population since event rates are not always reported per antithrombotic therapy group. Therefore, we additionally determined the event rates with the historical data of the NOTE patients for comparison. When compared with the historical data of the NOTE patients, previously using VKAs (2.4% for thromboembolism, 1.2% major bleeding), use of NOACs in adult Fontan patients results in similar rate of thromboembolism and major bleeding.

Compared with historical use of aspirin (6.2% for thromboembolism, 0% major bleeding), not surprisingly, thromboembolic event rate is lower and major bleeding rate is higher in our cohort. In summary, based on the comparison with the previous literature (1.9%–6.5% thromboembolism, 0.4%–3.8% major bleeding, 30.2% minor bleeding) and the historical data of this cohort (2.4% for thromboembolism, 0.8% major bleeding), the event rates under NOACs use (2.9% thromboembolism, 2.9% major bleeding, 15.8% minor bleeding) indicate that NOACs are safe at this short-term observation in Fontan patients.

Although earlier studies have shown that VKAs and aspirin reduce the rates of thromboembolism in Fontan patients, each has important limitations. For example, VKAs bear various disadvantages including interaction with many drugs, intercurrent illness and therapy non-compliance, which all have been associated with subtherapeutic or supratherapeutic INRs in Fontan patients.^4^ Furthermore, subtherapeutic INRs in Fontan patients have been shown to increase incidence of thromboembolism by 3.5–5.9 times compared with Fontan patients maintained in the therapeutic range.^13^ Furthermore, it is apparent from clinical practice that the cardiac lesion is not the only major problem for these patients, but also other issues such as the burden of medication use in their daily lives. The need for frequent monitoring interferes with these young patients’ social and working life, in addition to the possible stress caused by the daily variation in dose and the uncertainty if the INR is not in the correct range. Antiplatelet therapy does not have such limitations, but whether it has the optimal efficacy to prevent thromboembolism in Fontan patients is yet to be determined since the results on its efficacy varies among the studies, some showing same efficacy as warfarin,^5 6^ but also some showing inferior efficacy compared with warfarin.^2^ Also, it should be taken into account that one study observed 52% of aspirin-treated Fontan adults showing aspirin resistance, further questioning the practical implementation of aspirin in Fontan adults.^5^ Therefore, NOACs may be great alternatives to overcome limitations of VKAs and antiplatelet therapy if met with similar efficacy and safety on long term. However, one has to keep in mind that lack of INR monitoring in this predominantly young patients may lead to problems in adherence to NOACs on long term. Nonetheless, adherence data from the real-world studies in the general population with AA do show similar or even better adherence rates with NOACs than in VKA patients.^14^ Given the prospect of long-term use of thromboprophylaxis in adults with Fontan circulation, prescribers should always be aware of the risk of non-persistence and adverse effects of therapy and evaluate these issues at every follow-up.

Our study is limited by the heterogeneity of indications for NOAC use, confounding by indication, modest sample size, limited number of events over a relatively short-term follow-up, and the retrospective and observational designs. Furthermore, our rate of thromboembolism may be underestimated as subclinical thromboembolism may be missed due to lack of additional diagnostics such as ventilation–perfusion scan or CT at follow-up. Although half of this cohort used VKA previously, discrepancy in baseline characteristics with the other half, who did not use VKA previously, may limit the comparability of incidence rates in thromboembolism and major bleeding. Nonetheless, this worldwide study does provide the first promising evidence of efficacy and safety of NOACs in adults with Fontan circulation and offers solution to common research limitations (eg, inherent small sample size, retrospective design) in Fontan patients through international multicentre collaboration.

## Conclusions

In conclusion, NOACs are well tolerated and their efficacy and safety during short-term follow-up seem to be comparable to use of VKAs in adults with Fontan circulation. Longer term data are required to confirm such promising early results.
